# Etude cas-témoins des facteurs associés à l´insuffisance pondérale à la naissance au Centre Hospitalier de Kingasani, Kinshasa (République Démocratique Congo)

**DOI:** 10.11604/pamj.2021.38.94.16099

**Published:** 2021-01-27

**Authors:** Jean Claude Kaka Tshinzobe, Denise Kwango Ngaya

**Affiliations:** 1 Institut Supérieur des Techniques Médicales de Kinshasa, Section Santé Communautaire, Kinshasa, République Démocratique du Congo,; 2 Centre Hospitalier Kingasani, Service de Maternité, Kinshasa, République Démocratique du Congo

**Keywords:** Faible poids, nouveau-né, accouchement, Low weight, newborn, delivery

## Abstract

**Introduction:**

l´insuffisance pondérale à la naissance est considérée comme l´un des plus importants indicateurs de chances de survie d´un nouveau-né et un risque majeur de morbidité, de mortalité périnatale et infantile. L´objectif de cet article est d´étudier les facteurs associés à l´insuffisance pondérale à la naissance au Centre Hospitalier de Kingasani, à Kinshasa.

**Méthodes:**

une étude rétrospective de type cas-témoins a été réalisée. Les informations sur les parturientes et leurs enfants nés du 1^er^ janvier au 31 décembre 2016 ont été collectées dans le registre de la maternité du Centre Hospitalier Kingasani. Quatre cent cinquante-huit (458) cas (moins de 2500 grammes) ont été comparés aux 458 témoins (2500-4000 grammes). L´analyse multivariée a été faite à l´aide de la régression logistique binaire pour identifier les facteurs associés au faible poids de naissance.

**Résultats:**

en 2016, 3451 naissances vivantes ont été enregistrées et la prévalence de l´insuffisance pondérale a été estimée à 13,27%. Il a été trouvé, dans l´analyse bivariée, que la parité, le terme de grossesse, le type de grossesse et le sexe du nouveau-né sont des variables significativement associées à l´insuffisance pondérale à la naissance. Après ajustement sur les variables intégrées dans l´analyse multivariée, la parité, le terme de grossesse et le type de grossesse sont demeurés significativement associées à l´insuffisance pondérale à la naissance.

**Conclusion:**

de ces résultats, nous suggérons la promotion des études intégrant tous les paramètres impliqués dans la survenue de l´insuffisance pondérale à la naissance pour suivre l´évolution régulière de ce problème et des facteurs associés.

## Abstract (en)

**Introduction:**

low birth weight is considered one of the most important indicators for the survivability of a newborn and for a higher risk of morbidity, perinatal mortality and infant mortality. The purpose of this study was to analyse factors associated with low birth weight at the Kingasani Hospital in Kinshasa.

**Methods:**

we conducted a retrospective case-control study. The data about the information of the parturients and their children born from 1^st^ January to 31^st^ December 2016 were collected from the maternity register of the Kingasani Hospital Center. In this study 458 cases (less than 2500 grams) were compared to 458 controls (2500-4000 grams). Multivariate analysis was carried out using binary logistic regression in order to identify factors associated with low birth weight.

**Results:**

in 2016, 3451 live births were registered and the rate of underweight was estimated to 13.27%. Bivariate analysis showed that parity, the term of pregnancy, pregnancy type and infant’s sex were variables significantly associated with low birth weight. After adjusting for variables integrated in multivariate analysis, parity, the term of pregnancy and pregnancy type were still significantly associated with low birth weight.

**Conclusion:**

given these results, new studies of all the parameters involved in the occurrence of low birth weight are necessary in order to monitor the regular evolution of this issue and its associated factors.

## Introduction

Le poids à la naissance est un indicateur de l´état de santé à court et à long terme d´un nouveau-né [1]. Il prédit l´évolution pondérale d´un enfant dans son enfance. L´UNICEF définit l´insuffisance pondérale à la naissance comme le pourcentage de nouveaux nés pesant moins de 2500 grammes à la naissance [2]. De même, le faible poids à la naissance est défini par l´OMS comme le poids à la naissance inférieur à 2500 grammes, quel que soit l'âge gestationnel du nouveau-né. [1]. Un enfant est né avec un très faible poids à la naissance lorsqu´il pèse à la naissance moins de 1500 grammes. Un faible poids à la naissance est considéré comme l'un des plus importants indicateurs de chances de survie d'un nouveau-né, étant donné qu'un tel poids constitue un risque majeur de mortalité périnatale et infantile. Les bébés ayant un faible poids à la naissance courent plus de risques de connaître des problèmes de santé et de développement, dont des difficultés d'apprentissage, des déficiences auditives et visuelles, des problèmes respiratoires chroniques tels qu'asthme et des maladies chroniques plus tard au cours de leur vie [3].

Dans une étude réalisée en 2008 à Lubumbashi, Ntambue A *et al*. ont trouvé que les bébés de faible poids de naissance sont près de 16 fois plus à risque d´une mortalité périnatale et ceux de très faible poids de naissance courent 49 fois le risque d´une mortalité périnatale [4]. Dans le monde, l´insuffisance pondérale à la naissance contribue pour 60 à 80% à l´ensemble des décès néonatals. Selon l´OMS, chaque année, il est compté environ 20 millions de nourrissons dont le poids était insuffisant à la naissance et 96,5% d´entre eux naissant dans les pays en développement [5]. A cause de son impact sur la morbidité et la mortalité infantile, ainsi que de ses implications sur la santé à l´âge adulte, les experts en santé publique sont unanimes que l´insuffisance pondérale à la naissance constitue un problème majeur de santé publique [6]. D´après les statistiques présentées par l´UNICEF, l´insuffisance pondérale à la naissance représente respectivement 16%, 13% et 10% des naissances vivantes dans le monde, en Afrique subsaharienne et en RD Congo [2]. Les études antérieures en RDC et dans d´autres pays africains ont rapporté des prévalences de l´insuffisance pondérale à la naissance, allant de 6,3% à 13,6%. Parmi les principales causes incriminées du faible poids à la naissance, la prématurité et le retard de croissance intra-utérine sont les plus cités dans la littérature [7]. Les facteurs de risque qui déterminent l´insuffisance pondérale à la naissance ont été recherchés dans plusieurs études tant en Afrique qu´ailleurs. Dans le contexte africain, nous pouvons citer celle menée en milieu semi-rural de Kamina [RD Congo] par Bwana Kangulu I *et al*. dans laquelle les auteurs ont trouvé que l´âge maternel, la parité, la prématurité, la grossesse multiple et le non suivi des consultations prénatales sont des facteurs significativement associés au faible poids à la naissance [8]. Dans le même registre, l´étude transversale conduite par Kabore P *et al*. dans le Centre Nord du Burkina a identifié, comme facteurs significativement associés au petit poids de naissance, après ajustement pour les facteurs socio-économiques, la primiparité, les vomissements gravidiques, l´exécution de travaux champêtres, une charge de travail élevée en cours de grossesse et l´accouchement à domicile sans assistance [6]. Dans la présente étude, notre objectif est de rechercher des facteurs associés à l´insuffisance pondérale à la naissance dans un milieu hospitalier kinois en vue de proposer des pistes d´interventions pour la prévenir.

## Méthodes

L´étude a été réalisée au Centre Hospitalier Kingasani (CHK), situé dans la zone de santé de Kingasani, à l´Ouest de la province sanitaire de Kinshasa, en RD Congo. C´est une formation médicale opérant au sein du Bureau Diocésain des œuvres Médicales de Kinshasa. Le comité éthique de ce dernier a autorisé la réalisation de l´étude et a accordé son avis favorable quant aux aspects bioéthiques de la recherche.

Nous avons réalisé une étude rétrospective de type cas-témoins. Les informations sur les parturientes et leurs enfants nés du 1^er^ janvier au 31 décembre 2016 ont été collectées par une infirmière dans le registre de la maternité du CHK du 20 décembre au 19 février 2017.

Les cas (n = 458) sont tous les nouveaux nés vivants avec un poids inférieur à 2500 grammes, quels que soient le terme et le type de la grossesse. Les témoins (n = 458) sont des nouveaux nés présentant les mêmes caractéristiques que les cas, mais ayant pesé à la naissance entre 2500 et 4000 grammes. Chaque cas a été apparié à un témoin, né le même jour. Au total, la population d´étude s´élève à 916 nouveaux nés vivants. Les morts nés et les nouveaux nés transférés ont été exclus de l´étude.

Les variables suivantes ont été collectées: l´âge de la mère à l´accouchement, la parité, les antécédents de l´avortement, les antécédents de mortinatalité, l´âge gestationnel, le poids de bébé à la naissance, le sexe de bébé et le type de grossesse. Les informations recueillies ont été saisies sur une feuille Excel 2007 et traitées avant d´être importées pour l´analyse statistique à l´aide du logiciel SPSS 20. La description de la population d´étude a été faite à travers les statistiques usuelles: les pourcentages des modalités des variables qualitatives ont été présentés dans un tableau approprié; les moyennes et les écart-types des variables quantitatives ont été calculés. L´analyse bivariée a été effectuée à l´aide du test de khi-deux de Pearson pour étudier séparément les variables indépendantes potentiellement associés à l´insuffisance pondérale à la naissance, la variable dépendante. Les pourcentages du faible poids à la naissance ont été comparés dans les différentes modalités des variables indépendantes collectées. Les conditions d´application ont été vérifiées au préalable. L´analyse multivariée a été faite à l´aide de la régression logistique binaire, pour identifier après ajustement, sur les facteurs associés au faible poids de bébé à la naissance identifiés au niveau de l´analyse bivariée. Le test d´Hosmer et Lemeshow a été effectué pour étudier l´adéquation des résultats aux données. Le seuil de signification de 5% a été utilisé dans toutes les analyses tant bivariées que multivariées.

## Résultats

Pendant la période d´étude, 3451 naissances vivantes ont été enregistrées au CHK. La prévalence de l´insuffisance pondérale, en 2016, a été estimée à 13,27%. Les caractéristiques, les antécédents obstétricaux des accouchées et les caractéristiques des nouveaux nés ont été comparés dans les deux groupes. Les résultats de cette comparaison sont présentés dans le Tableau 1. L´insuffisance pondérale à la naissance est fréquente chez les nouveaux nés de sexe masculin (54,8%), les mères adultes (69,9%), les primipares (40,8%), celles ayant accouché avant terme (77,9%) et celles ayant donné naissance aux grossesses multiples (17,9%). En analyse bivariée, il a été trouvé que la parité, le terme de grossesse, le type de grossesse et le sexe des nouveaux nés sont des variables significativement associées à l´insuffisance pondérale à la naissance.

**Tableau 1:** OR bruts de l´insuffisance pondérale à la naissance (IPN) en fonction des caractéristiques, antécédents obstétricaux des accouchées et caractéristiques des nouveaux nés (analyse bivariable)

Variables	IPN	PN	OR brut (IC à 95%)	p
	n(%)	n(%)		
**Age des accouchées**				0,09
˂20 ans (n=133; 14,5%)	78(17,0)	55(12,0)	0,67(0,46-0,98)	
20-34 ans (n=655; 71,5%)	320(69,9)	335(73,1)	1	
35 ans et plus (n=128; 14%)	60(13,1)	68(14,9)	1,08(0,74-1,58)	
**Parité des accouchées**				0,009
1 (primipare) (n=326; 35,6%)	187(40,8)	139(30,3)	0,60(0,44-0,81)	
2-3 (paucipare) (n=327; 35,7%)	146(31,9)	181(39,6)	1	
4-6 (multipare) (n=227; 24,8%)	107(23,4)	120(26,2)	0,90(0,64-1,27)	
7-11 (grande multipare) (n =36; 3,9%)	18(3,9)	18(3,9)	0,80(0,40-1,60)	
**Terme de grossesse**				<0,001
Avant terme (n=442; 48,7%)	357(77,9)	85(18,6)	15,51(11,23-21,42)	
A terme (n=466; 51,3%)	101(22,1)	373(81,4)	1	
**Type de grossesse**				<0,001
Grossesse multiple (n=98; 10,7%)	82(17,9)	16(3,5)	6,02(3,46-10,47)	
Grossesse unique (n=818; 89,3%)	376(82,1)	442(96,5)	1	
**Antécédent d´avortement**				0,83
Oui (n=108; 11,8%)	55(12)	53(11,6)	0,95(0,64-1,43)	
Non (n=808; 88,2%)	403(88)	405(88,4)	1	
**Antécédent de mortinatalité**				0,50
Oui (n=38; 4,1%)	21(4,6)	17(3,7)	0,80(0,41-1,54)	
Non (n=878; 95,9%)	437(95,4)	441(96,3)	1	
**Sexe**				0,01
Masculin (n=466; 50,9%)	251(54,8)	215(46,9)	1,37(1,05-1,79)	
Féminin (n=450; 49,1%)	207(45,2)	243(53,1)	1	

PN = Poids normal

Le Tableau 2 expose les résultats de l´analyse multivariée par la régression logistique binaire. Après ajustement sur les variables intégrées dans l´analyse, le résultat renseigne que la parité, le terme de grossesse et le type de grossesse sont demeurés significativement associées à l´insuffisance pondérale à la naissance. Mais, il a été constaté qu´en termes de risque de donner naissance à un bébé de faible poids, les primipares présentent un risque réduit comparativement aux multipares et aux grandes multipares.

**Tableau 2:** OR ajustés de l´insuffisance pondérale en fonction de la parité, du terme de grossesse et du type de grossesse

Variables	OR ajustés [IC à 95%]	p
**Parité**		0,02
1 (primipare)	0,54 [0,36-0,81]	
2 à 3 (paucipare)	1	
4 à 6 (multipare)	0,66 [0,43-1,03]	
7 à 11 (grande multipare)	0,72 [0,29-1,80]	
**Terme de grossesse**		<0,001
Avant terme	16,71 [11,87 -23,53]	
A terme	1	
**Type de grossesse**		<0,001
Grossesse multiple	7,78 [4,09-14,80]	
Grossesse unique	1	

Variables non significatives: âge des accouchées et sexe des nouveaux nés

## Discussion

En réalisant une étude rétrospective de type cas-témoins, notre objectif était la recherche des facteurs qui sont associés à l´insuffisance pondérale à la naissance au CHK. Au total, 916 parturientes ont été incluses dans l´étude dont 458 cas et 458 témoins. Un cas a été apparié à un témoin. Le même appariement a été constaté dans d´autres études notamment celles de Camara B *et al*. [9] qui ont étudié 178 cas et 178 témoins, de Mabiala-Babela JR *et al*. [10] comparant 488 cas à 488 témoins. Tandis que les auteurs suivants ont apparié plus d´un témoin à un cas: 6 témoins à un cas par Bwana Kangulu I *et al*. [8], soit 69 cas contre 414 témoins; 4 témoins à un cas par Hassoune S *et al*. [11] soit 30 cas contre 120 témoins et 2 témoins à un cas par Demelash H *et al*. [12] soit 136 cas contre 272 témoins.

Dans les critères d´inclusion des sujets dans l´étude, nous avons retenu la prématurité et les grossesses multiples, à l´instar d´autres chercheurs [8]. Par contre, certains auteurs [9, 10, 13] ont exclu soit les grossesses multiples soit les naissances prématurées [14], ou les deux à la fois [6, 7]. Pendant la période de l´étude, la prévalence de l´insuffisance pondérale à la naissance au CHK a été estimée à 13,27%. Cette prévalence est supérieure à celle de toute la RD Congo qui équivaut à 10%, mais elle avoisine celle de l´Afrique subsaharienne, estimée à 13%. Des études menées pendant une année dans certaines villes ont trouvé des prévalences de FPN divergentes: dans la banlieue de Dakar au Sénégal, 10,7% en 1996 [9]; dans la ville de Harare, 19,9% en 1998 [15]; à Moshi au Nord de Tanzanie, 13.6% en 2001 [16]; au Centre Nord du Burkina Fasso, 15,8% des naissances à terme en 2003 [6], dans la ville d´Antananarivo, 12,9% en 2004 [17], dans la commune de Tori Bossito au Bénin, 9,1% en 2007 [18], dans une localité rurale de Gambie, 10,5% en 2008 [19], au Benin City au Nigeria, 6,3% en 2015 [20].

Au terme de l´étude, il a été trouvé, qu´après ajustement sur les variables intégrées dans l´analyse, la parité, le terme de grossesse et le type de grossesse sont des variables significativement associées à l´insuffisance pondérale à la naissance. Plusieurs études ont mentionné des résultats similaires [6, 8, 10, 14, 17, 18, 21, 22]. Ces auteurs ont constaté également que le poids des bébés à la naissance augmente avec la parité des mères. En d´autres termes, le faible poids de naissance diminue proportionnellement avec la parité des parturientes.

L´accouchement avant terme et les naissances multiples ont été trouvés, dans notre étude, comme des variables fortement associées au faible poids de naissance. A ce sujet, notre résultat corrobore ceux d´autres chercheurs [8, 11, 13, 19, 20, 23-25]. L´âge maternel n´a, cependant, pas été constaté comme facteur de risque de l´insuffisance pondérale à la naissance dans notre série des données. Les résultats de certaines études antérieures en Ethiopie, au Nigeria et à Malawi convergent aux nôtres [26-28]. Nous ne pouvons clore cette étude sans en mentionner une limite importante, à savoir, la source des données utilisée dans cette étude. En effet, le registre de la maternité du CHK n´a pas renseigné sur les événements survenus et les comportements de consommation des mères au cours de la grossesse, les consultations prénatales, le poids et la taille des parturientes. Et pourtant, ces variables sont reconnues dans la littérature comme des facteurs de risque potentiels de l´insuffisance pondérale à la naissance et méritaient d´être intégrées dans l´analyse des données [29].

## Conclusion

Pendant la période d´étude, il a été trouvé qu´au CHK, la prévalence du déficit pondéral à la naissance était de 13,27%. Au terme de l´ajustement sur les variables intégrées dans l´analyse, la parité, le terme de grossesse et le type de grossesse sont des facteurs demeurés significativement associés à l´insuffisance pondérale à la naissance. En définitive, nous suggérons la promotion des études intégrant tous les paramètres impliqués dans la survenue de l´insuffisance pondérale à la naissance pour suivre l´évolution régulière de ce problème de santé publique et des facteurs associés récurrents. La connaissance de ces derniers, modifiables ou non, permettra un suivi prénatal ciblé des gestantes à risque de faible poids de naissance.

### Etat des connaissances sur le sujet

- L´insuffisance pondérale à la naissance est un indicateur de chances de survie d´un nouveau, de risque de morbidité et mortalité périnatale;
- Les caractéristiques des mères, son mode de vie pendant la grossesse, son histoire gynécologique influencent le poids du nouveau-né.

### Contribution de notre étude à la connaissance

- L´étude a trouvé l´influence majeure de la prématurité et des grossesses multiples dans la survenue du faible poids de naissance;
- Elle a suggéré également l´intégration, dans la recherche, de tous les facteurs potentiels qui pourraient concourir à l´insuffisance pondérale à la naissance;
- La non-intégration de tous les facteurs potentiels risque de ne pas tenir compte de certains paramètres dans les stratégies de prévention.

## Remerciements

Les auteurs remercient Madame Marie-Laure Charkaluk, de l´Institut Catholique de Lille, pour la lecture critique de l´article et les observations suggérées dans la version finale de l´article.
